# Body composition changes differ by gender in stomach, colorectal, and biliary cancer patients with cachexia: Results from a pilot study

**DOI:** 10.1002/cam4.1665

**Published:** 2018-07-03

**Authors:** Saunjoo L. Yoon, Oliver Grundmann, Joseph J. Williams, Lucio Gordan, Thomas J. George

**Affiliations:** ^1^ Department of Biobehavioral Nursing Science College of Nursing University of Florida Gainesville FL USA; ^2^ Department of Medicinal Chemistry College of Pharmacy University of Florida Gainesville FL USA; ^3^ Sunshine Integrative Health Gainesville FL USA; ^4^ Florida Cancer Specialists & Research Institute Gainesville FL USA; ^5^ Division of Hematology & Oncology Department of Medicine College of Medicine University of Florida Gainesville FL USA

**Keywords:** bioelectrical impedance analysis, body composition, cachexia, colorectal cancer, gender, phase angle

## Abstract

Few studies have examined the possibility that cachexia may affect men and women differently. This pilot study assessed gender differences in body composition in stomach, colorectal, and biliary cancer patients with cachexia. A sample of 38 participants (Female: Male = 17:21, mean age 57.4 years) were included if they were undergoing chemotherapy and experienced weight loss of 5% or more over a 6‐month period. Bioelectrical impedance analysis (BIA) was applied to measure body composition. Phase angle (PA) and levels of extra‐/intracellular water (ECW; ICW) were determined. Data were analyzed first by gender and then compared to age‐ and gender‐matched healthy controls from the NHANES‐III dataset. PA was lower (*P *<* *.01) in both genders compared with healthy controls, and PA was lower in female patients compared with male patients (*P *=* *.03). Male cancer patients with lower PA also had lower ICW levels compared with healthy controls (*r *=* *.98, *P *<* *.01). For female patients, PA and ICW were negatively correlated (*r *=* *.897, *P *<* *.01). A lower ECW/ICW ratio was highly correlated (*r *=* *.969 for men, *r *=* *.639 for women) with increased PA in cancer patients. ICW changes are gender‐specific in patients with GI cancer. ECW/ICW ratios and PA may be suitable surrogate markers for gender‐specific changes in cell composition and health status.

## 1 INTRODUCTION

Research has consistently shown that body composition and fat metabolism differ between men and women,^1^ and that men have a higher percentage of fat‐free mass (FFM), while women naturally have more fat mass (FM). It is also well established that fatty acid metabolism is gender‐specific in healthy populations. However, to date, it is not clear whether male and female patients with cancer cachexia demonstrate a similar pattern of body composition and phase angle (PA) changes, especially when compared to their age‐ and gender‐matched healthy controls as known reference values.

Cancer cachexia is a comorbid disorder that affects over 50% of all patients with cancer and is estimated to have a 20%‐60% 1‐year mortality among all cancer types.^2^ Weight loss as an indicator of cachexia is highest among patients with gastrointestinal (GI) cancers followed by lung and other respiratory cancers.^3^ Other symptoms of cachexia, in addition to unintentional weight loss, may include fatigue, anorexia, sarcopenia, increased inflammatory markers, decreased muscle strength, and lean muscle mass depletion with or without loss of fat mass.^4^ Cachexia often progresses as a result of cancer metabolism and chemotherapy, which contribute to systemic inflammation, physical obstruction (eg, esophageal cancer, intestinal cancer), and pain.^5^, ^6^ Sarcopenia is the loss of lean muscle mass which naturally occurs as we age but is accelerated by inflammation and cachexia.^7^ Inflammation is a hallmark of cancer cachexia and often leads to changes in cell integrity and water balance due to shifts in blood pH.^8^

**Figure 1:**
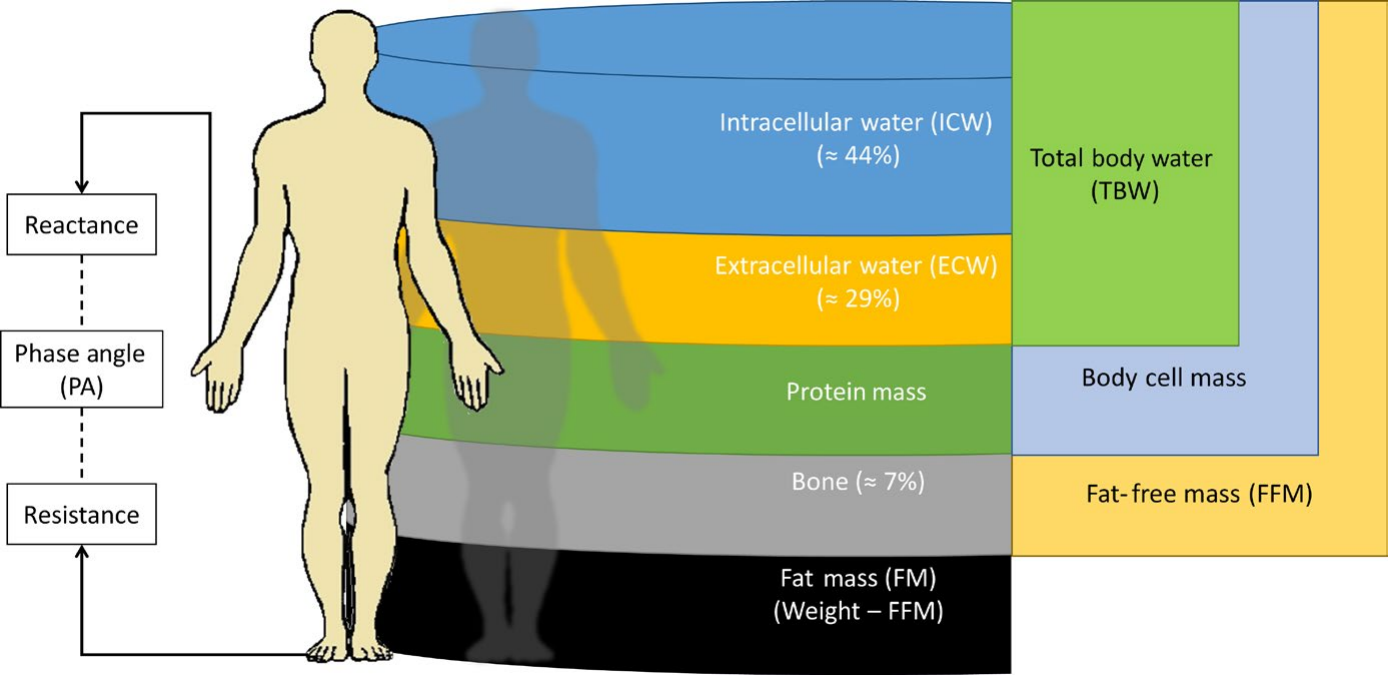
Bioelectrical impedance analysis (BIA) measurements. How the raw measures of reactance and resistance can be used to derive phase angle (PA) and body composition measures used in this study

To date, a variety of biomarkers such as pro‐inflammatory markers have been used to diagnose cachexia, and body composition variables and PA have been frequently used as prognostic variables for survival in cancer patients with cachexia.^9^, ^10^ PA, which is a composite indicator of the resistance and reactance representing fluid distribution in cells and cell wall integrity,^11^ can be measured via bioelectrical impedance analysis (BIA). Indeed, BIA enables a noninvasive measurement of body composition and PA, as well as derived measurements of FM, FFM, extra‐/intracellular water (ECW; ICW), and total body water (TBW) (Figure 1).^11^ Because cancer cachexia is defined by both weight and lean muscle loss, body composition analysis is a promising way to detect nutritional depletion, muscle wasting, and changes in these parameters over time.^11^

Thus, the purpose of this study was to investigate for the first time gender differences in body composition with a focus on ECW, ICW, FM, FFM, and PA of both male and female stomach, colorectal, and biliary cancer patients with cachexia compared to a healthy reference population from the NHANES‐III dataset.^12^ A similar comparative study using the same dataset was conducted on patients with end‐stage renal disease, and although it compared body composition measures, it did not make a distinction between genders.^13^ The proposed study is novel because it uses BIA as a primary measurement tool as well as controls for the age of patients, type and stages of cancer, and study size.

## 2 METHODS

The data presented here represent the baseline comparative measurements from participants enrolled in a pilot two‐group, single‐blind, randomized controlled study to examine the effect of an intervention on appetite in GI cancer patients with cachexia. The study was approved by the Institutional Review Board (IRB‐01, IRB201400340) of the institution where the study was conducted prior to study commencement. Participants were recruited from 2 oncology outpatient clinics in town. This study included all participants (N = 38) who completed the baseline data collection prior to initiation of the intervention and who were considered eligible for the study.

Inclusion criteria—The study included patients who: (1) were 21 years and older; (2) were able to communicate in English; (3) agreed to follow the research protocol; (4) had received a medical diagnosis of gastrointestinal cancer (eg, gastric, biliary, small intestine, or colorectal); (5) were starting or continuing chemotherapy at the time of screening for participants; and (6) had a 5% or more weight loss over a preceding 6‐month period. All patients were receiving standard of care, which could include measures to stimulate appetite such as steroids or appetite‐promoting medications. Exclusion criteria—The study excluded patients who: (1) planned to have surgical procedures at the time of recruitment; (2) were scheduled to receive radiation therapy alone or in addition to chemotherapy during the study period; (3) underwent surgery during the study or in the month prior to the study and did not have chemotherapy scheduled postsurgery; (4) had any comorbidities that could affect the interpretation of study findings (eg, HIV, AIDS, Alzheimer's disease, movement disorder, acute myocardial infarction within last 3 months, hepatitis); (5) had open burn sites or infected wounds; (6) were diagnosed with esophageal cancer with a swallowing difficulty in mechanical nature; (7) had an uncorrected, mechanical digestive obstruction, or inability to tolerate enteral nutrition; (8) had a diagnosis of pancreatic adenocarcinoma; and (9) had a life expectancy of less than 6 months, as assessed by an attending physician.

Baseline BIA data of 38 participants (M:F = 21:17) were extracted and compared to matched reference values taken from the NHANES‐III dataset of healthy Americans.^12^ Two‐sided Student's *t* test and simple linear regression with *P* < .05 were used for statistical comparisons. All data were analyzed using Microsoft Excel 2013 (version 15.0, Microsoft, Seattle, WA) and GNU PSPP (http://www.gnu.org/software/pspp/).

### 2.1 Measures and instruments

#### 2.1.1 Body composition

Body composition of study participants with GI cancer was compared to a healthy reference population data retrieved from the NHANES‐III dataset. Bioelectrical impedance analysis (BIA) using the ImpediMed Imp^™^ SFB7, (ImpediMed Ltd., Eight Mile Plains, QLD, Australia) was applied to measure body composition (more information about the device, ImpediMed Imp^™^ SFB7 can be found on the website; https://www.impedimed.com/wp-content/products/SFB7/SFB7_CA_Brochure.pdf). ImpediMed Imp^™^ SFB7, which has a single channel tetrapolar configuration with a touch screen, is an objective tool used to measure FM, FFM, ICW, ECW, and PA,^14^, ^15^, ^16^, ^17^ and is a reproducible, noninvasive, and validated device in patients with cancer.^17^

#### 2.1.2 Demographic information

Demographic information, including cancer type, stage, and treatment, age, gender, education, marital status, ethnicity, and insurance type/status, was obtained from participants after signing an informed consent and before conducting the first intervention.

#### 2.1.3 Weight, height, and body mass index

The weight and height of participants were measured at baseline. Body mass index (BMI) was calculated using the weight and height measurements, and results were entered into the BIA device to calculate body composition.

## 3 RESULTS

Demographic characteristics: A sample of 38 participants (Female: Male = 17:21) enrolled in the study (Table 1). Mean age was 57.4 years old (Female: Male = 55.1 year: 59.2 year) with 12 patients (31.6%) being 65 years and older, 18 patients (47.4%) between 50 and 64 years, and 8 patients (21.0%) between 21 and 49 years old. A majority of patients (32/38, 84.2%) were either diagnosed with an advanced (3 or 4) cancer stage or the stage remained undetermined. The PA in female patients with cancer was significantly lower than that of male patients with cancer (*P *=* *.03), whereas the PA in age‐matched healthy controls did not significantly differ (*P *=* *.12) (Table 2). However, PA was significantly (*P *<* *.01) lower in both genders (F = 4.05°, M = 4.81°) compared with their respective healthy controls (F = 7.06°, M = 7.30°) (Table 2). A subgroup analysis of patients 65 years and older indicated no significant difference in PA between gender in patients with cancer (*P *=* *.73) or their control groups (*P *=* *.46).

**Table 1:** Baseline demographics and characteristics of cancer patients

Baseline demographics	Total (N = 38)	Female	Male
Age (min‐max)	57.4 (27‐75)	55.1 (27‐74)	59.1 (28‐75)
Gender (F:M)	38	17	21
Ethnicity/Race			
Caucasian	28	11	17
African American	7	5	2
Hispanics	2	0	2
Others	1	1	0
Marital status			
Single/never married	4	3	1
Married	21	6	15
Divorced	9	5	4
Widowed	4	3	1
Education years (Mean: 12.8)			
8‐11th grades	4	0	4
12th grade	10	5	5
13th or higher	24	12	12
Employment			
Employed	8	3	5
Disabled	10	4	6
Retired	12	4	8
Student	1	1	0
Not working for other reasons	7	5	2
Cancer diagnosis			
Colorectal	27	13	14
Stage 2	5	3	2
Stage 3	4	2	2
Stage 4	8	4	4
Stage undetermined	10	4	6
Gastric	7	1	6
Stage 1	1	0	1
Stage 3	1	0	1
Stage 4	3	1	2
Stage undetermined	2	0	2
Biliary	4	3	1
Stage 4	1	1	0
Stage undetermined	3	2	1
BMI (min‐max)	26.3 (19.0‐34.8)	26.7 (19.0‐34.8)	25.9 (19.1‐33.9)

**Table 2:** Body composition parameters by gender and condition. Means displayed with standard deviations, SD, in parenthesis. Determination of significance via *t* test with *P *<* *.05

BIA parameter	Cancer cachexia patients			Age‐matched healthy controls		
	Female (SD)	Male (SD)	Significance (*P*)	Female (SD)	Male (SD)	Significance (*P*)
Phase angle	4.05 (0.76)	4.81 (1.24)	*.03*	7.06 (0.46)	7.30 (0.75)	.12
Total body water	45.95% (4.41)	54.57% (5.87)	*<.01*	46.36% (0.89)	55.52% (0.51)	*<.01*
Extracellular water	46.06% (1.94)	48.36% (3.41)	*.02*	47.02% (0.40)	41.70% (0.41)	*<.01*
Intracellular water	53.94% (1.94)	51.64% (3.41)	*.02*	52.98% (0.40)	58.30% (0.41)	*<.01*
Fat mass	37.23% (6.03)	25.45% (8.02)	*<.01*	37.88% (1.20)	25.40% (0.68)	*<.01*
Fat‐free mass	62.77% (6.03)	74.55% (8.02)	*<.01*	62.12% (1.20)	74.60% (0.68)	*<.01*
Body mass index	26.74 (4.38)	25.85 (3.67)	.38	28.44 (0.98)	27.01 (0.63)	*<.01*

Italics: significant difference at p < 0.05.

**Figure 2:**
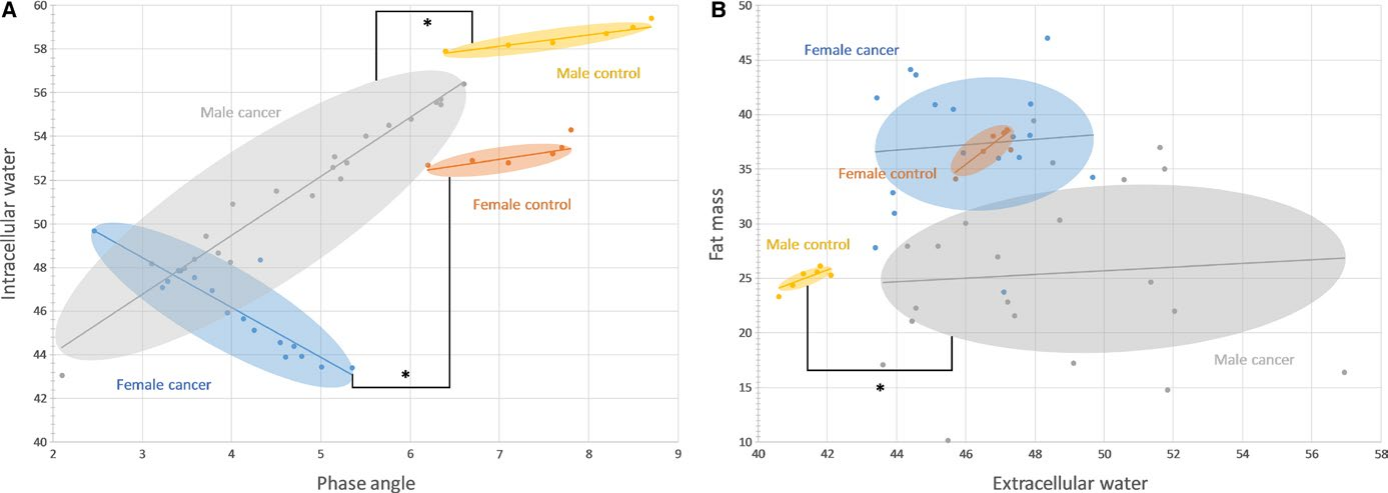
A, Phase angle versus intracellular water (%) by gender and condition. Healthy controls were age matched to corresponding gender. Determination of significance between cancer and control group was based on correlation analysis with *P *<* *.05. B, Extracellular water (%) vs fat mass (%) by gender and condition. Healthy controls were age matched to corresponding gender. Determination of significance between cancer and control group was based on correlation analysis with *P *<* *.05

A direct comparison between genders revealed that for TBW, ECW, ICW, FM, and FFM both patients with cancer and healthy controls presented with a significant difference (*P *=* *.02 for ECW and ICW in patients with cancer, *P *<* *.01 for all other parameters) (Table 2). The baseline BMI was significantly higher for healthy women than that of men (*P < *.01) which was not the case in the patients with cancer (*P *=* *.38). Correlation between ICW and PA revealed that male cancer patients with lower PA also had lower ICW compared to that of their healthy controls (*r* = .98, *P *<* *.01) (Figure 2A). For women, the correlation between PA and ICW was negative, and higher ICW corresponded to lower PA in patients with cancer (*r* = .897, *P *<* *.01). In general, patients with cancer 65 years and older reflected the same significant changes by gender compared to their age‐matched healthy controls.

This was not reflected in the corresponding healthy female control group (Figure 2A). Women in both the cancer and the healthy control population had significantly higher FM (*P *<* *.01) compared to that of their respective male counterparts (Figure 2B). However, only male patients with cancer had a significantly higher ECW compared to that of their healthy controls, while the FM remained unchanged between the male groups (*r *=* *.005, *P *>* *.05) (Figure 2B). Both female cancer and healthy control populations did not present with a difference in the correlation between ECW and FM (*r *=* *.07, *P *>* *.05) (Figure 2B).

**Figure 3:**
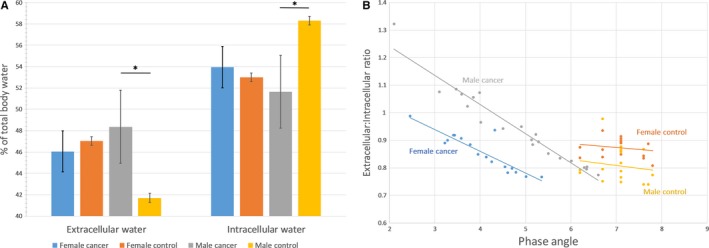
A, Extracellular water (%) and intracellular water (%) by gender and condition. Columns represent means with bars showing standard deviations. Determination of significance via *t* test with *P *<* *.05. B, Phase angle versus extracellular: intracellular ratio by gender and condition. Healthy controls were age‐matched to corresponding gender. Correlation coefficient for each group: male cancer: *r *=* *.969, female cancer: *r *=* *.894, male control: *r *=* *.153, female control: *r *=* *.192

A statistically significant shift from ICW to ECW (*P *<* *.01) in men was observed compared to that of age‐matched healthy controls (Figure 3A), whereas female patients with cancer did not show a statistically significant shift (*P *=* *.054) in the opposite direction from ECW to ICW (Figure 3A) relative to their healthy controls (Figure 3A and Table 2). The ratio between the extracellular and intracellular water content correlated significantly with the PA for both male and female patients with cancer (*r *=* *.969 and *r* = .894, *P *<* *.010) (Figure 3B). In contrast, both healthy age‐matched control groups did not present with this correlation (*r *=* *.153 for male and *r *=* *.192 for female, *P *>* *.05) (Figure 3B).

**Figure 4:**
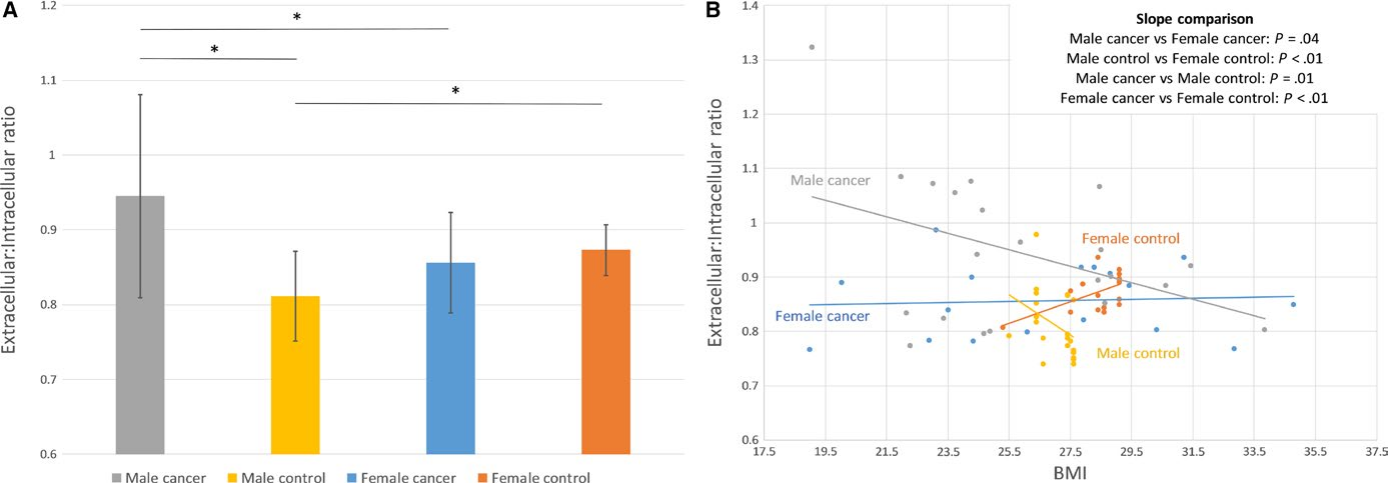
A, Extracellular: intracellular ratio by gender and condition. Columns represent means with bars showing standard deviations. Determination of significance via *t* test with *P *<* *.05. B, Phase angle versus extracellular: intracellular ratio by gender and condition. Healthy controls were age matched to corresponding gender. Determination of significant slope differences between cancer and control groups was based on correlation and two‐sided *t* test analysis with *P *<* *.05

A direct comparison of the ECW to ICW ratio between all 4 groups indicates significant differences between male patients with cancer and their respective controls, male and female patients with cancer, and healthy male and female controls (Figure 4A). Correlation between BMI and ECW to ICW ratio revealed a distinguishing gender difference with female cancer patients presenting with no correlation between BMI and ECW to ICW ratio, while their controls indicate an increase in ECW to ICW ratio with increased BMI (Figure 4B). The opposite was observed for male cancer and control patients with a less significant decrease in ECW to ICW ratio change as BMI increases in male cancer patients compared with their healthy controls (Figure 4B). All 4 groups were significantly different from one another.

A majority of patients (71%) were diagnosed with colorectal cancer. Stage 4 colorectal cancer group had a large enough sample size to compare gender differences for various BIA measurements (Table 3). Male cancer patients presented with significant higher FFM compared to female colorectal cancer patients (*P *=* *.04), while all other measurements were not significantly different. The PA tended to be higher for male colorectal cancer patients compared with females but did not reach significance (*P *=* *.06).

**Table 3:** Comparison of body composition parameters by gender and cancer stage in colorectal cancer patients. Only stage 4 colorectal cancer had sufficient sample size in both genders to be compared. Means displayed with standard deviations, SD, in parenthesis. Determination of significance via *t* test with *P *<* *.05

Body composition	Colorectal cancer stage		
	Stage 4		
	Female (SD), N = 4	Male (SD), N = 4	Significance (*P*)
Phase angle	3.31 (0.62)	4.51 (0.84)	.06
Extracellular water	47.83% (1.53)	48.71% (2.17)	.53
Intracellular water	52.17% (1.53)	51.29% (2.17)	.53
Fat‐free mass	62.53% (2.85)	74.00% (8.34)	***.04***
Body mass index	28.51 (4.80)	28.32 (1.94)	.95
ECW:ICW ratio	0.92 (0.06)	0.95 (0.08)	.52

Italics and bold: significant difference at p < 0.05.

## 4 DISCUSSION

As body weight alone does not reflect the true health status of patients with cancer, BIA should be an integral part of caring for patients with cancer and monitoring the status of their health. Overall, the results of this study show that cachexia affects women and men differently, especially with respect to changes in fat mass and levels of intracellular water. Reference values of PA can differ by gender and age. PA values are lower in female gender and old age compared to their gender counterparts. Reference values of PA in males and females between 50 and 59 were 7.31 ± 0.89 and 6.55 ± 0.87, respectively.^18^ Phase angle has been regarded as the best indicator for overall health and predictor for survival in patients with cancer to date. In addition, both genders in our pilot study had significantly reduced PA compared to that of healthy controls, and these results agree with the literature,^16^, ^18^ which shows that patients with cancer cachexia lose muscle mass and suffer from depleted nutrition and increased inflammation. A subset analysis of older patients (≥65 years old) and their respective healthy controls indicates that most findings of this study apply to older patients except PA, which may not be significantly different between genders as metabolism slows down and overall health declines.

Intracellular water serves as an important measure of cell wall integrity and overall cellular health in regard to systemic inflammation. With respect to gender differences, female patients had higher ICW but similar FM compared to those of their healthy controls, indicating a shift of ECW to ICW within FFM as well as a lower degree of inflammation than that experienced by male patients with cancer.^19^, ^20^ Similarly, because male patients had significantly lower ICW and significantly higher FFM compared to those of female patients, the lower ICW may indicate more aggressive inflammation.^20^ This appears to accelerate with advanced cancer stages although the sample size in this study was small and can only be applied to patients with colorectal cancer.

The correlation between PA and ICW may serve as an indicator of overall health and a predictor of survival. For example, male patients with cancer experienced a positive correlation between PA and ICW, which suggests that cell wall integrity increases along with ICW, leading to a higher PA and longer survival in patients with cancer.^11^, ^21^ In contrast, female patients with cancer experienced a negative correlation between PA and ICW, and lower ICW was associated with higher PA, which contradicts the idea that a higher PA leads to longer survival.^22^ The lower ICW in females can be potentially linked to stress and inflammation with loss of cell integrity and muscle mass atrophy.^20^ There is also some indication that sex hormone differences may account for variable expression levels of specific gastrointestinal cancers such as for gastric cancer where expression of estrogen receptors reduces the risk while androgen receptors will increase the risk of GI cancers.^23^ Another hypothesis may relate to the specific cancer stage and chemotherapy that the patient has received. Because of the small sample sizes in each cancer stage and the wide spread for chemotherapy cycles, only a preliminary analysis of patients with colorectal cancer in stage 4 was possible pointing to FFM was a potential indicator for gender differences.

In advanced cancer stages, increased inflammation often leads to poor hydration status and cellular stress. In addition, chemotherapy is not specific to cancer cells and often burdens the whole body leading to immune system impairment and subsequent pro‐inflammatory markers.^24^ Several chemotherapies can cause dehydration to which women may be more prone given a lower TBW than men and thus making them more sensitive to inflammatory processes as mentioned above.^25^

Finally, changes to the ECW/ICW ratio and FFM may have occurred because cell wall integrity and cellular hydration imbalance are caused by cancer and its treatment. Typically, the ratio of ECW/ICW serves as an indicator of cell wall integrity and cell health and implies that essential cellular functions are maintained and the tissue is able to uphold osmotic pressure and ion concentration gradients.^26^, ^27^ The ratio of ECW/ICW also serves as an indicator of cell hydration status; indeed, dehydrated cells are small in size and can contribute to protein catabolism in skeletal muscles^28^ and the ECW/ICW ratio is considered to be useful in assessing muscle strength in elderly women.^29^ Because of its role as an indicator of cell wall integrity and cellular hydration, the ratio of ECW/ICW has been cited both as a surrogate marker for PA during evaluation of patients’ overall health as well as for localized tissue health.^30^ Significantly, in this study, a decreased ECW/ICW ratio was correlated with an increase in PA for both male and female patients with cancer, which suggests that patients with lower ECW/ICW ratios had better health than patients with higher ECW/ICW ratios. This correlation was also present for the BMI in male patients with cancer, while the BMI of female patients with cancer did not correlate with the ECW/ICW ratio. While the male control group presented with the same trend, the female control group indicated a positive correlation between ECW/ICW ratio and BMI which remains unexplained. In contrast, there was no apparent correlation between PA and ECW/ICW ratios for the healthy controls for either gender, which may not be apparent in a healthy population.

Overall, the results of this pilot study indicate PA as a suitable surrogate marker for changes in cell composition and health status but point to its limitations in distinguishing between gender‐specific responses. BIA is a feasible, noninvasive, clinical tool that can be used to quantify and monitor cachexia in patients with GI cancer. Considering the prevalence of cachexia in patients with cancer and the limited research on gender‐specific BIA changes, more research that examines why female patients with cancer experienced a decrease in ICW is warranted. Notably, the findings of this study are limited by the small sample size; thus, future studies with a larger sample size will likely decrease variability and provide a better comparison with the healthy control group. Additional studies to determine whether clinical interventions that impact cancer cachexia can rely on PA and the ECW/ICW ratio as surrogate markers are ongoing. Future research in this area will also help optimize both prevention and intervention in patients with cancer who are at risk or are already developing signs of cachexia.

## CONFLICT OF INTEREST

The authors declare no conflict of interest.

## ETHICS APPROVAL AND CONSENT TO PARTICIPATE

The study was approved by the Institutional Review Board (IRB‐01, IRB201400340) at the University of Florida. Written informed consent was obtained from all subjects before the start of the study.
